# Associations between accelerometer-measured physical activity and sedentary behaviour with physical function among older women: a cross-sectional study

**DOI:** 10.1186/s12889-024-19270-7

**Published:** 2024-07-02

**Authors:** Yanyu Lu, Qingqian Li, Wenbo Wang, Litao Du, Qiang He, Si Chen, Xianliang Zhang, Yang Pan

**Affiliations:** 1 School of Physical Education, Shandong University, Jinan, China; 2 Jinan Engineering Polytechnic, Jinan, China; 3 Zaozhuang Vocational College of Science and Technology, Zaozhuang, China; 4 School of Nursing and Rehabilitation, Cheeloo College of Medicine, Shandong University, Jinan, China

**Keywords:** Activity pattern, Joint effect, Physical function, Sedentary behaviour

## Abstract

**Background:**

This study aimed to investigate the relationships between accelerometer-measured physical activity (PA) and sedentary behaviour (SB) with physical function (PF) among older Chinese women in the community.

**Methods:**

The present study comprised 1,113 community-dwelling older females, with an average age of 65 ± 2 years. We employed a linear regression analysis to investigate the relationship between patterns of PA and SB with PF. PA variables consisted of total PA time, bouted PA time (a continuous PA that lasts equal to or more than 10 min), and sporadic PA time (a continuous PA that lasts less than 10 min). SB variables included total SB time, 30-min bout of SB (a continuous SB that lasts equal to or more than 30 min), and 60-min bout of SB (a continuous SB that lasts equal to or more than 60 min). PF variables comprised handgrip strength (HGS), one-legged stance test with eyes closed (OLSTEC), usual walking speed (UWS), maximum walking speed (MWS) and chair-stand time (CT). To explore the joint effects of moderate-to-vigorous-intensity PA (MVPA) and SB on PF, we divided the duration of SB and MVPA participation in older women into different combinations: low MVPA & high SB, low MVPA & low SB, high MVPA & high SB, high MVPA & low SB.

**Results:**

The study revealed a significant association between 30-min bout of SB and CT, which remained after adjusting for total MVPA time (*P* = 0.021). Both total MVPA and bouted MVPA were found to be positively associated with better UWS, MWS, CT, and PF Z-score. When the combination of low MVPA & high SB was used as a reference, the regression coefficients for PF ascended by 1.32 (*P* < 0.001) in the high MVPA & high SB group and by 1.13 (*P* < 0.001) in the high MVPA & low SB group.

**Conclusions:**

A significant association was observed between poorer lower limb function and prolonged, uninterrupted SB in older women, rather than with the total SB time. Concurrently, the insufficient engagement in MVPA may also be a crucial factor contributing to poorer PF in older women. Engaging in longer durations and higher intensity of PA, such as bouts of MVPA lasting a minimum of 10 min or longer, may contribute to better PF.

**Supplementary Information:**

The online version contains supplementary material available at 10.1186/s12889-024-19270-7.

## Background

As individuals age, there is a tendency towards a reduction in physical activity (PA) and an increase in sedentary behaviour (SB) [1]. This trend is one of the key reasons for the decline in physical function (PF) [2, 3]. The decline in PF not only leads to a reduced quality of life in older adults [4, 5], but also rises the risk of falls and can lead to disability and loss of independence in severe cases [6, 7], placing a heavy burden on individuals, families and society. In contrast with men, the decrease in bone muscle mass, muscle strength, and PF is more pronounced in older women [8, 9]. Even more concerning is that older women are more susceptible to sarcopenia and related disease [10, 11]. In addition, older women have higher rates of disability [12], long-term care needs and medical expenses than men [13, 14]. These risks are strongly connected with PF, which means that identifying modifiable risk factors for preventing fall in PF is especially important for older females.

PA and SB are crucial factors affecting PF and the health status of elderly individuals [15, 16], and their relationship with PF in older women deserves further study. The relationships between PA and SB with PF are currently unclear, which impedes the design of effective interventions. Firstly, it is inconsistent among previous studies as to whether PA affects the association between SB and PF [17–19]. Secondly, the majority of research on the relationship between PA and PF has focused on the cumulative duration of moderate-to-vigorous-intensity PA (MVPA), with limited attention given to the cumulative patterns of MVPA. In 2020, the World Health Organization (WHO) revised its PA guidelines to omit the prior recommendation of at least 10 min of MVPA at a time. The update underscored that any volume of activity confers benefits relative to inactivity [20]. This revision serves to enhance the public’s comprehension of the potential advantages associated with various patterns of PA, including sporadic MVPA (duration < 10 min). Substantial research indicates that sporadic MVPA correlates with decreased instances of metabolic syndrome [21], fear of fall [22], frailty [23]and mortality [24]in older adults. Nevertheless, the relationship between sporadic MVPA and PF has only been explored in one study to date. Furthermore, the PF data in their study were obtained through self-reporting, which could introduce potential biases [25]. It has been observed that older adults tend to have a more negative view of their own health and functional abilities as they age [26]. This may result in lower levels of self-reported PF, which may in turn affect the accuracy of the results of studies based on self-reports [27]. Additionally, numerous studies have demonstrated that light intensity PA (LPA) can have a beneficial impact on the health of a larger population [28–30], yet few studies have incorporated LPA into PF research. A review of the literature reveals that there is no consensus on the relationship between LPA and PF in a few studies. Some studies have indicated that LPA is related to PF [31, 32], while others have suggested that LPA is not related to PF [33, 34]. For older individuals, it may be more feasible to engage in LPA than MVPA.

Consequently, in light of these gaps, this study aimed to explore the relationships between patterns of PA and SB with PF in older women to provide more accurate PA recommendations to improve PF in older females.

## Methods

### Participants

The cross-sectional data was sourced from the baseline surveys of the PA and Health in Older Women Study (PAHIOWS). PAHIOWS was a community-based study conducted in a coastal city in Shandong Province, China, aimed at exploring the relationship between PA and SB and the health status of older females. A total of 1,370 participants aged between 60 and 70 years were included in the PAHIOWS. These participants were able to communicate freely, were without cognitive impairment (Mini-Mental State Examination [35](MMSE) scores > 18), and had signed informed consent forms. This research was approved by the ethics committee of the School of Nursing and Rehabilitation, Shandong University, China (2020-R-067). All participants provided written informed consent.

### Measurement of sedentary behaviour and physical activity variables

A triaxial accelerometer (ActiGraph wGT3X-BT, Pensacola, FL, USA) was employed to collect the participants’ PA and SB data [36]. The instructions were to wear the accelerometer on the subject’s hip for seven consecutive days, with the exception of periods of sleep, bathing, and swimming. While participants wore the accelerometer, they received two phone follow-ups to ensure compliance with the accelerometer-wearing protocol. Participants were included in the final analysis if they wore the accelerometer for a minimum of 10 h each day over at least four validation days [37].

The data were recorded in 60-s epochs and analyzed using ActiLife software, version 6.13.4 (https://www.actigraphcorp.com/). Nonwear time was defined as 90 consecutive min or longer of zero-intensity counts, with no more than 2 min of counts between 0 and 100 [38]. In this study, SB was defined as < 100 count per minute (CPM), LPA was defined as 100–1951 CPM and MVPA was defined as > 1952 CPM. The cut-point of the MVPA corresponds approximately to the energy expenditure of a walking speed of 4 km/h [39]. The three cut-points were based on those developed by Freedson et al. [40] and have been validated in numerous studies of older women [41–44]. Meanwhile, the PA and SB variables were exported in order to identify specific patterns. The SB variables include: (i)Total SB time (Average daily sedentary time); (ii)30-min bout of SB (each time consisting of 30 consecutive minutes or more of SB); (iii)60-min bout of SB ( each time consisting of 60 consecutive minutes or more of SB). The PA variables include: (i)Total PA time (Average daily PA time); (ii)Bouted PA (each time consisting of 10 consecutive minutes or more of PA); (iii)Sporadic PA (any PA accumulated in < 10 min).

### Measurement of physical function variables

This study employed a comprehensive assessment of PF in older women who met the inclusion criteria, utilising five validated performance tests [45]. The tests were administered one-on-one by trained staff at a fitness monitoring center. Before each test, participants were informed of about the specific requirements and asked if they were able to complete it. Individuals who self-reported an inability to perform the tests were excluded from the study. If participants confirmed their ability to undertake the tests, the staff closely monitored their condition, ready to provide assistance or discontinue the tests if necessary. The aforementioned tests encompass the following:

Handgrip strength (HGS)(ACMEWAY(Beijing) Health Technology, China): HGS testing is a validated and feasible bedside method that is currently the most commonly used method in clinical practice to measure upper-body strength [46, 47]. Participants were asked to stand with their arms parallel to the body and squeeze an electronic hand dynamometer (in kg) with their dominant hand as hard as possible on two occasions, with the highest value being recorded as the measurement.

One-legged stance test with eyes closed (OLSTEC) : The OLSTEC is a common method for assessing body balance and stability, and it is a predictor of fall risk in older adults [48, 49]. During the test, participants were instructed to close their eyes, maintain a parallel position of their arms to their bodies, elevate one foot off the ground and maintain balance. The timer starts when the foot is lifted, and the participant stands on one leg as long as possible (up to 120 s). In the event that the stationary foot is displaced, or the lifted foot contacts the ground, the timer is terminated. The dominant leg was subjected to two trials, with the most favourable outcome being selected for analysis.

5-m walk test: The 5-metre walk test has been demonstrated to be a reliable and valid indicator of mobility in older adults [49]. The participants were requested to complete two tests on a 7-metre track, one at their usual walking speed (UWS) and one at their maximum walking speed (MWS). The testers recorded the time taken by the participants to reach the 5-metre line on each occasion and subsequently analysed the measurements from both times.

Chair-standing time(CT): This test is used to assess lower limb functional strength [50, 51] and has been identified as a feasible, reliable, and effective measure for the prediction of falls in older adults in the community [52]. The participants were instructed to cross their hands in front of their chest, ascend from a stool without armrests to a fully standing position as quickly as possible, and then return to a seated position as soon as possible, which was repeated five times. The total time taken to complete the five repetitions was recorded by the testers using a stopwatch.

A standardised PF composite score (Z-score) was calculated by summing the individual Z-scores of the five tests [53]. Since a lower Z-score of CT, USW, and MWS indicates superior physical ability, the three Z-values were recorded as opposite numbers. The cumulative sum of these five Z-values served as the comprehensive continuous measurement for PF.

### Measurements of other variables

Considering the effect of other factors on result, we collected data of potential confounding factors, which were shown to be associated with the variable of PF [54–57]. Sociodemographic characteristics including age, living alone (yes or no) were collected using face-to-face interview. Income (currency: CNY, unit: monthly/yuan; income options include ≤ 1000 yuan (approximately 137 dollars), 1001–2000 yuan, 2001–3000 yuan, 3001–4000 yuan, and > 4000 yuan) and the number of chronic diseases (assessed by self-reported history of medical diagnosis, consist of diabetes, heart disease, stroke, cancer, chronic lung, etc., defined as 0, 1, 2, 3 or more diseases) were measured. The Chinese version of the Athens Insomnia Scale (AIS) was used to assess sleep quality, with a total score ranging from 0 to 24 points, with higher scores representing more severe insomnia [58]. Nutritional status was measured using the Mini Nutritional Assessment Short Form (MNA) with a total score of 30, and lower scores indicate worse nutritional status [59]. Cognitive function was measured using the MMSE, with a total score ranging from 0 to 30, where lower scores were regarded as indicative of lower cognitive levels [35]. The body mass index (BMI) was derived by measuring the height and weight of subjects by a body composition analyzer (MC-180, TANITA, JAPAN) and then using the following formula: BMI = weight (kg)/height^2^ (m^2^).

### Statistical analysis

Descriptive data were presented as means ± standard deviation (SD) for continuous variables and as frequency (percentages) for categorical variables. A linear regression analysis was employed to investigate the relationship of SB and PA with PF indicators and PF Z-score, respectively. In order to more precisely assess the effects of incremental changes in PA and SB on PF, new variables for SB and PA were developed. The variables represent the daily increments in time spent in SB, LPA, and MVPA. Specifically, the SB variable was configured to increase by 60 min per day in order to simulate an increase in SB. Concurrently, the LPA variable was adjusted to incorporate an additional 30 min per day to account for elevated levels of LPA, while the MVPA variable was set to incorporate an extra 10 min per day to signify increased MVPA [60, 61]. The following three models were used to adjust for confounding factors: Model 1 was adjusted for age and accelerometer daily wear time. Model 2 was additionally adjusted for Model 1 variables plus confounding factors including BMI, living alone, income, number of chronic diseases, AIS score, MNA score, and MMSE score. Model 3 additionally adjusted for corresponding accelerometer variables according to the specific independent variable. Model 3a was adjusted for total MVPA time to all SB variables and total LPA time, and Model 3b was adjusted for total SB time to total MVPA time. To investigate the independent associations between different PA patterns and PF, bouted PA and sporadic PA were adjusted for each other: Model 3c was adjusted for Model 2 variables plus total SB time, and additionally adjusted for bouted MVPA and sporadic MVPA to each other; Model 3d was adjusted for Model 2 variables plus total MVPA time, and additionally adjusted for bouted LPA and sporadic LPA to each other [60]. The variance inflation factor (VIF) for all variables were calculated to detect the presence of collinearity. In the fully adjusted Model 3, VIF below 3 which was considered acceptable [62].

To investigate the joint effects of MVPA and SB on PF, we divided the daily time spent in MVPA and SB among older women into tertiles. Based on the lowest and highest tertiles, participants’ activity combinations were categorized into four types: Low MVPA & High SB (MVPA < 23.2 min and SB ≥ 570.8 min); Low MVPA & Low SB (MVPA < 23.2 min and SB < 494.7 min); High MVPA & High SB (MVPA ≥ 38.4 min and SB ≥ 570.8 min) and High MVPA & Low SB (MVPA ≥ 38.4 min and SB < 494.7 min). Finally, linear regression analysis was performed using Models 1 and 2 described above. Regression coefficients and 95% confidence intervals (CI) represent all linear regression results. A value of *P* < 0.05 was considered significant, with *P* < 0.01 deemed highly significant. All computations were performed using Stata version 17.0.

## Results

### Participant characteristics

Of all 1370 older females, 148 women had insufficient accelerometer wear time, 97 women did not complete the questionnaire assessment, and 12 women did not finish PF assessment. The final analysis included data from 1,113 older females. The characteristics of the study population are presented in Table 1. The survey findings indicated that the average age of older women was 65 years old, 11.2% lived alone, 13.8% had three or more chronic diseases, and 50.9% earned between 3,001 and 4,000 yuan. In accordance with the established criteria for BMI classification (overweight defined as BMI > 25 kg·m^− 2^), the mean BMI of the study population was 25.4 kg·m^− 2^, indicating that the majority of participants were overweight.

**Table 1:** Participant characteristics (*n* = 1113)

	M ± SD or *n* (%)
Age, years	64.9 ± 2.8
Living alone, n (%)	125(11.2)
BMI, kg·m^− 2^	25.4 ± 3.3
Income, n (%)	
≤ 1000 yuan	35(3.1)
1001–2000 yuan	106(9.5)
2001–3000 yuan	182(16.4)
3001–4000 yuan	567(50.9)
> 4000 yuan	223(20.0)
Number of chronic diseases, n (%)	
0	387(34.8)
1	382(34.3)
2	191(17.2)
≥ 3	153(13.8)
AIS score, point	1.0 ± 2.3
MNA score, point	13.3 ± 1.1
MMSE score, point	26.7 ± 1.4
Sedentary behaviour variables, min/day	
Total SB time	548.3 ± 117.7
30-min bout of SB	172.2 ± 97.3
60-min bout of SB	76.4 ± 67.5
Physical activity variables, min/day	
Total MVPA time	32.7 ± 19.2
Bouted MVPA	11.8 ± 13.1
Sporadic MVPA	20.9 ± 11.0
Total LPA time	307.3 ± 70.5
Bouted LPA	181.9 ± 74.5
Sporadic LPA	125.4 ± 23.0
Physical function variables	
Handgrip strength, kg	24.1 ± 4.5
OLSTEC, s	9.7 ± 9.5
UWS, m/s	3.8 ± 0.6
MWS, m/s	2.8 ± 0.4
CT, s	8.0 ± 2.1
Z-score, point	0.±3.1

Note: Data shows MEAN ± SD or N (%). BMI, Body mass index; CNY, ChiNaYuan;

AIS, Athens Insomnia Scale; MNA, Mini Nutritional Assessment Short Form;

MMSE, Mini-Mental State Examination; SB, Sedentary Behaviour;

MVPA, Moderate-to-Vigorous-intensity Physical Activity; Bouted, duration ≥ 10 min;

Sporadic, duration<10 min; LPA, Light intensity Physical Activity;

OLSTEC, One-legged stance test with eyes closed;

UWS, Usual walking speed; MWS, Maximum walking speed; CT, chair-standing time.

### Associations between patterns of physical activity, sedentary behaviour and physical function

Table 2 presents the final results (Model 3) of the associations between PA and SB with PF. Further details on Models 1 and 2 can be found in the supplementary material, Tables S1-S6. According to Table 2, among all SB variables, only 30-min bout of SB showed a significant association with CT (*P* = 0.021) after adjusting for total MVPA time. Regarding PA, both total MVPA time and Bouted MVPA were significantly associated with UWS, MWS, CT, and the PF Z-score. Specifically, for each increment of 10 min for total MVPA time, UWS decreased by 0.19 m/s (95% CI: -0.25, -0.14, *P* < 0.001), MWS decreased by 0.12 m/s (95% CI: -0.16, -0.75, *P* < 0.001), CT decreased by 0.44s(95% CI:-0.65,-0.23, *P* < 0.001) and the PF Z-score increased by 0.94 (95% CI: 0.63, 1.23, *P* < 0.001). Similarly, for each increment of 10 min for Bouted MVPA, UWS decreased by 0.07 m/s (95% CI: -0.10, -0.04, *P* < 0.001), MWS decreased by 0.03 m/s (95% CI: -0.05, 0.02, *P* < 0.001), CT decreased by 0.11s(95% CI:-0.20,-0.01, *P* = 0.024) and the PF Z-score increased by 0.25 (95% CI: 0.11, 0.39, *P* < 0.001). Moreover, there was no significant association between total LPA time, bouted LPA and sporadic LPA with PF in Model 3. Notably, HGS and OLSTEC had no association with any variables of SB and PA.

**Table 2:** Linear regression of the associations between PA, SB variables, and PF (model 3)

The PA and SB variables	HGS		OLSTEC		UWS		MWS		CT		Z-score	
	B (95% CI)	*P*	B (95% CI)	*P*	B (95% CI)	*P*	B (95% CI)	*P*	B (95% CI)	*P*	B (95% CI)	*P*
Per 60 min increment of												
Total SB time	-0.03(-0.21, 0.27)^a^	0.826	-0.13(-0.65, 0.39)^a^	0.632	0.01(-0.02, 0.04)^a^	0.515	0.00(-0.02, 0.20)^a^	0.099	0.09(-0.02, 0.20)^a^	0.099	-0.09(-0.25, 0.07)^a^	0.266
30-min bout of SB	-0.09(-0.31,0.12)^a^	0.390	-0.25(-0.72,0.21)^a^	0.283	0.00(-0.03,0.03)^a^	0.894	0.01(-0.01,0.03)^a^	0.510	0.11(0.01,0.21)^a^	0.021*	-0.12(-0.26, 0.02)^a^	0.093
60-min bout of SB	-0.14(-0.44,0.16)^a^	0.345	-0.31(-0.96,0.33)^a^	0.344	0.01(-0.03,0.04)^a^	0.765	0.00(-0.02,0.03)a	0.765	0.09(-0.05,0.23)^a^	0.197	-0.13(-0.33, 0.07)^a^	0.202
Per 10 min increment of												
Total MVPA time	0.36(-0.10,0.82)^b^	0.125	0.33(-0.66,1.32)^b^	0.518	-0.19(-0.25,-0.14)^b^	< 0.001**	-0.12(-0.16,-0.75)^b^	< 0.001**	-0.44(-0.65,-0.23)^b^	< 0.001**	0.94(0.63, 1.23)^b^	< 0.001**
Bouted MVPA	0.01(-0.19,0.22)^c^	0.889	-0.09(-0.54,0.36)^c^	0.693	-0.07(-0.10,-0.04)^c^	< 0.001**	-0.03(-0.05,0.02)^c^	< 0.001**	-0.11(-0.20,-0.01)^c^	0.024*	0.25(0.11, 0.39)^c^	< 0.001**
Sporadic MVPA	0.10(-0.03,0.22)^c^	0.131	0.19(-0.08,0.46)^c^	0.159	-0.00(-0.02,0.01)^c^	0.706	-0.01(-0.02,0.01)^c^	0.334	-0.04(-0.10,0.02)^c^	0.153	0.08(-0.00, 0.17)^c^	0.065
Per 30 min increment of												
Total LPA time	-0.01(-0.13,0.11)^a^	0.826	0.06(-0.20,0.32)^a^	0.632	-0.01(-0.02,0.01)^a^	0.515	-0.00(-0.02,0.01)^a^	0.451	-0.05(-0.10,0.01)^a^	0.099	0.05(-0.03,0.13)^a^	0.266
Bouted LPA	0.00(-0.12,0.12)^d^	0.992	0.09(-0.17,0.36)^d^	0.484	-0.00(-0.02,0.01)^d^	0.551	-0.00(-0.02,0.01)^d^	0.421	-0.05(-0.11,0.00)^d^	0.073	0.05(-0.03, 0.14)^d^	0.193
Sporadic LPA	0.24(-0.18,0.66)^d^	0.259	0.62(-0.29,1.52)^d^	0.182	0.00(-0.05,0.05)^d^	0.967	-0.01(-0.05,0.03)^d^	0.580	-0.13(-0.32,0.06)^d^	0.175	0.20(-0.08,0.48)^d^	0.158

PA, Physical Activity; SB, Sedentary Behaviour; HGS, hand grip strength; OLSTEC, One-legged stance test with eyes closed; UWS, usual walking speed; MWS, maximum walking speed; CT, chair-standing time;

B, Regression Coefficient; CI, confidence intervals; MVPA, Moderate-to-Vigorous-intensity Physical Activity; LPA, Light intensity Physical Activity; Bouted, duration ≥ 10 min; Sporadic, duration<10 min.

The superscripts a, b, c, d, and d represent Model 3a, Model 3b, Model 3c, and Model 3d;

Model 3a: Model 2 + total MVPA time;

Model 3b: Model 2 + total SBtime;

Model 3c: Model 2 + total SB time, and additionally adjusted for bouted MVPA and sporadic MVPA to each other;

Model 3d: Model 2 + total MVPA time, and additionally adjusted for bouted LPA and sporadic LPA to each other.

*P-value < 0.05, **P-value < 0.01.

### The joint associations of sedentary behaviour and moderate-to-vigorous-intensity physical activity categories with physical function measures

The analysis showed that MVPA levels moderated the relationship between SB and PF. The results of the combined effect of SB and MVPA on PF are shown in Table 3. In Model 2, we found that PF was significantly associated with both the high MVPA group compared to the Low MVPA & High SB combination. The regression coefficient of the High MVPA & High SB combination increased by 1.32 (95% CI: 0.83, 1.82, *P* < 0.001), and the regression coefficient of the High MVPA & Low SB combination increased by 1.13 (95% CI: 0.63, 1.63, *P* < 0.001). Notably, no association with PF was found for the Low MVPA & Low SB combination compared to the Low MVPA & High SB combination.

**Table 3:** The joint associations of SB and MVPA categories with PF measures

Different combinations of PA and SB	B (95% Cl)			
	Model 1	*p*	Model 2	*p*
Low MVPA & High SB	1(Reference)		1(Reference)	
Low MVPA & Low SB	0.74(-0.12, 1.59)	0.091	0.08(-0.46, 0.62)	0.781
High MVPA & High SB	2.28(1.45, 3.10)	<0.001**	1.32(0.83, 1.82)	< 0.001**
High MVPA & Low SB	2.21(1.45, 2.97)	<0.001**	1.13(0.63, 1.63)	< 0.001**

B, Regression Coefficient; CI, confidence intervals;

SB, Sedentary Behaviour; MVPA, Moderate-to-Vigorous-intensity Physical Activity;

Model 1: Adjusting accelerometer daily wear time and age;

Model 2: Model 1 + BMI, Living alone, Income, Number of chronic diseases, AIS score, MNA score, and MMSE score;.

**P-value < 0.01.

## Discussion

Overall, our study in Model 3 demonstrates a significant association between 30-min bouts of SB and CT. In terms of PA, both total MVPA time and bouted MVPA were associated with better UWS, MWS, CT, and PF Z-score. However, all LPA variables show no significant association with any PF variables. Compared to the combination of low MVPA & high SB, high MVPA level combinations exhibit a significant improvement in PF Z-score regardless of the level of SB.

The subjects of this study were older women residing in the community. Their average sedentary time was 9.1 h per day, less than the 9.4 h previously reported for most older adults [63]. This may be attributed to the fact that in our community, residents engage in social interactions with their neighbours on occasion, which may contribute to a reduction in sedentary time. Previous studies have shown that total SB time and 60-min bout of SB in older adults were associated with poor performance on OLSTEC and CT [64]. Nevertheless, our findings do not fully corroborate the previously proposed association. Specifically, our results indicate that in older women, 30-min bout of SB was associated with worse CT manifestations, while other SB variables had no significant association with PF indicators and Z-score. This suggests that total SB time may not be the main influencing factor, but longer continuous sedentary bout patterns could pose a risk to specific PF indicators, especially lower limb function. It is likely that gender is a significant factor in this divergent outcome. Previous studies have revealed significant differences in PF between men and women [65–67]. For example, Gennuso et al. [67]. discovered that there was no significant association between 20, 40, and 60-min bout of SB and short physical performance battery score in the female sample. However, a significant association was observed in the male sample. This indicates that gender may play a moderating role in the relationship between SB and PF. Meanwhile, their study also reported that total SB time was not associated with PF in both male and female samples, consistent with our findings. Additionally, the participants in our study exhibited fewer instances of SB lasting 60 min or more, which represented only 14% of the total daily sedentary time on average. Approximately half of the older female participants engaged in less than one bout of SB lasting 60 min or more per day. In contrast, 30-min bout of SB was significantly more prevalent, accounting for 31.5% of the total sedentary time, nearly twice as much as the 60-min bout of SB. Notwithstanding the absence of a significant association between 60-min bout of SB and PF observed in our study, it cannot be ruled out that such prolonged periods of SB may adversely affect the PF of older women. In summary, our study indicates that it was the prolonged, uninterrupted patterns of SB, rather than the total SB time, that were more strongly linked to the decline in lower-extremity function among older adults. Subsequent research should take into account potential gender differences and further investigate the beneficial effects of reducing continuous SB on improving PF in older adults.

The analysis of MVPA and PF revealed a significant positive association between total MVPA time and PF enhancement, which was consistent with the results of both male and female samples [19, 68, 69]. Furthermore, our study highlights the benefits of bouted MVPA. More specifically, the PF regression coefficient increased by 0.25 for every 10 min/day increase in bouted MVPA. The results of this study are consistent with those of a previous investigation which demonstrated that both total time spent in MVPA and bouts of at least 10 min in duration were associated with improved PF in older women [70]. Sporadic MVPA was an important component of the total MVPA time. However, our study did not find an association between sporadic MVPA and PF. The reason why no association between sporadic MVPA and PF was found may be that our study sample had relatively healthier PF levels and that sporadic MVPA was not sufficient to improve PF. Thus, the effect of sporadic MVPA on PF needs to be applied to more people in the future to verify whether sporadic MVPA can improve lower PF levels. Based on our findings, we currently recommend that healthy older women should participate in MVPA lasting 10 min or more at a time to gain health benefits on PF. Moreover, a comprehensive examination of the data revealed a noteworthy phenomenon: a substantial proportion of the participants (70%) demonstrated compliance with the WHO recommendation of engaging in at least 150 min per week of MVPA. The high compliance rate may be attributed to the pervasive participation of older women in community square dancing activities across China. Square dancing is a popular group activity characterised by an upbeat rhythm and easily learned choreography. The dissemination of new steps and choreographic patterns is expedited by the guidance provided by lead dancers, which subsequently encourages greater participation among older adults. An interventional study has demonstrated that regular participation in square dancing can significantly improve cognitive function, reduce symptoms of depression, and enhance the quality of life in elderly individuals with mild cognitive impairment [71]. The results of our investigation indicate that community-based square dancing may play a significant role in enabling elderly Chinese women to achieve or even exceed the PA levels recommended by the WHO.

Although LPA was more likely to be involved relative to MVPA, all LPA variables were not associated with PF in this study. This result is consistent with the results of several previously published studies on total LPA time and PF [18, 33, 70, 72]. In a study by Izawa et al. [14], an association was identified between LPA and MWS among 181 older adults with a mean age of 73.4 ± 4.8 years. The finding indicate that LPA may enhance mobility in seniors who are unlikely to withstand high-intensity activities. The authors point out, however, that the limited size of the sample limits the generalizability of their findings. The results of our current research indicate that LPA was unlikely to be an effective intervention for maintaining or improving PF in healthy older adults. Furthermore, our analyses did not reveal any significant associations between the PA variables measured and either HGS or OLSTEC. It is likely that this finding can be attributed to the fact that individuals participating in this study demonstrated superior levels of PF on both tests. In accordance with the 2019 Asian Sarcopenia Working Group Consensus, a female handgrip strength under 18 kg signifies low HGS, whereas 18 kg and above is considered normal HGS [73]. It is crucial to highlight that the average HGS among the group of older women participating in this study was 24 kg, which is significantly higher than the threshold for normal HGS as defined by the aforementioned consensus from the 2019 Asian Sarcopenia Working Group. Furthermore, a meta-analysis that consolidated of data from 18 studies demonstrated that the OLSTEC test yielded an average duration of 8.29s for individuals aged 60 to 64, and 7.15s for those aged 65 to 69, among which the average performance of Chinese elderly samples was 8.05s [74]. In contrast to the aforementioned groups, the older women in our study achieved a mean duration of 9.73s on the balance test, which was significantly superior to the performances of all the referenced comparison groups. Consequently, the pronounced enhancements in handgrip strength and balance abilities observed among our sample of older women indicate that they may have attained a level of PF that sufficiently masks the potential benefits typically associated with structured PA.

In order to explore the joint effect of PA and SB on PF, participants’ activity combinations were divided into four categories: low MVPA & high SB, low MVPA & low SB, high MVPA & high SB, and high MVPA & low SB. The results of the analysis indicated that combination of high MVPA & high SB and high MVPA&low SB were independently associated with the improvement of PF in older females, indicating that regardless of the level of SB, higher levels of MVPA were related to the advancement of PF. A previous study that included male samples demonstrated that high SB & low MVPA and low SB & low MVPA were associated with poorer PF [75]. Their study further supported the notion that the detrimental impact on PF was primarily due to insufficient MVPA. Therefore, even for individuals who accumulate a high amount of sedentary time, maintaining an adequate level of MVPA can still confer health benefits, particularly in relation to PF. This insight was of value to older women who may be required to sit for extended periods due to occupational or lifestyle factors. It highlighted the significance of regular engagement in MVPA as a key factor in maintaining their functional health.

### Innovations and limitations

In the present study, accelerometers were worn at the hip to assess PA and SB among elderly women aged between 60 and 70 years. The hip has been validated as the optimal single placement for an accelerometer for the measurement of PA intensity. This methodology effectively minimized the burden on participants and enhanced the feasibility of the study. Consequently, the implementation of this methodology led to a notable enhancement in the precision of data collection, accompanied by a reduction in the potential for subjective bias. This, in turn, served to reinforce the reliability and accuracy of the study’s findings. Moreover, our research investigated the associations between diverse intensities and patterns of PA with PF in elderly women, as well as conducted a thorough analysis of the joint effects of PA and SB on PF. This provided a comprehensive perspective on how different intensities and patterns of PA independently or collectively affect PF. Notably, our study revealed for the first time the association between bouted LPA, sporadic LPA, and PF, contributing novel insights to the field of LPA research.

Despite these strengths, it is important to acknowledge several limitations. Firstly, the efficacy of the accelerometer in discerning body postures (i.e., standing, sitting, lying) may be constrained. Secondly, the cross-sectional design of the study precluded the establishment of causal relationships. Furthermore, the sample was drawn from a single city in China, which may not be sufficiently representative of the broader population of Chinese elderly individuals, limiting the generalisability of the findings. Finally, it is possible that the participants in this study were relatively healthy and more physically capable, which may have resulted in a selection bias towards individuals with better health statuses. In light of these limitations, future research should consider employing a longitudinal study design to explore more deeply the causal relationships between PA and SB patterns and PF in elderly women. Furthermore, it would be beneficial to expand the sample scope to encompass a more diverse population, while aiming for more precise measurement of SB and PA, in order to ensure the broad applicability of the study results.

## Conclusions

A significant association was observed between poorer lower limb function and prolonged, uninterrupted SB in older women, rather than with the total SB time. Concurrently, the insufficient engagement in MVPA may also be a crucial factor contributing to poorer PF in older women. Engaging in longer durations and higher intensity of PA, such as bouts of MVPA lasting a minimum of 10 min or longer, may contribute to better PF.

### Electronic supplementary material

Below is the link to the electronic supplementary material.

Supplementary Material 1

## Data Availability

The datasets used and analyzed during the current study are available from the corresponding author on reasonable request.

## Abbreviations

PA: Physical Activity
SB: Sedentary Behaviour
PF: Physical Function
MVPA: Moderate-to-Vigorous-intensity Physical Activity
WHO: World Health Organization
LPA: Light intensity Physical Activity
PAHIOWS: Physical Activity and Health in Older Women Study
OLSTEC: One-legged Stance Test with Eyes Closed
UWS: Usual walking speed
MWS: Maximum walking speed
CT: Chair-standing Time
CPM: Counts Per Minute
AIS: Athens Insomnia Scale
MNA: Mini Nutritional Assessment Short Form
MMSE: Mini-Mental State Examination
BMI: Body Mass Index
CI: Confidence intervals
VIF: Variance Inflation Factor
