# Supplementary material for: Associations between accelerometer-measured physical activity and sedentary behaviour with physical function among older women: a cross-sectional study
Source: BMC Public Health. 2024 Jul 2;24:1754. doi: 10.1186/s12889-024-19270-7 (PMC11218370; doi:10.1186/s12889-024-19270-7)
Supplement: Supplementary file 1 — Supplementary Material 1 [file 12889_2024_19270_MOESM1_ESM.docx]

| The PA and SB variables | B (95 % CI) | | | | | |
| --- | --- | --- | --- | --- | --- | --- |
|  | Model 1 | P | Model 2 | P | Model 3 | P |
| Per 60 min increment of |  |  |  |  |  |  |
| Total SB time | -0.06(-0.28, 0.16) | 0.608 | -0.05(-0.27, 0.17) | 0.670 | -0.03(-0.21, 0.27)^a^ | 0.826 |
| 30-min bout of SB | -0.12( -0.33,0.09) | 0.255 | -0.13(-0.34, 0.08) | 0.221 | -0.09(-0.31,0.12)^a^ | 0.390 |
| 60-min bout of SB | -0.18(-0.47,0.12) | 0.236 | -0.18(0.48,0.11) | 0.215 | -0.14(-0.44,0.16)^a^ | 0.345 |
| Per 10 min increment of |  |  |  |  |  |  |
| Total MVPA time | 0.18(-0.23,0.59) | 0.382 | 0.34(-0.08,0.76) | 0.114 | 0.36(-0.10,0.82)^b^ | 0.125 |
| Bouted MVPA | 0.01(-0.19,0.21) | 0.940 | 0.05(-0.15,0.25) | 0.647 | 0.01(-0.19,0.22)^c^ | 0.889 |
| Sporadic MVPA | 0.06(-0.06,0.18) | 0.375 | 0.09(-0.03,0.22) | 0.132 | 0.10(-0.03,0.22)^c^ | 0.131 |
| Per 30 min increment of |  |  |  |  |  |  |
| Total LPA time | 0.02(-0.10,0.14) | 0.763 | 0.00(-0.12,0.12) | 0.991 | -0.01(-0.13,0.11)^a^ | 0.826 |
| Bouted LPA | 0.00(-0.10,0.11) | 0.990 | -0.02(-0.13,0.08) | 0.664 | 0.00(-0.12,0.12)^d^ | 0.992 |
| Sporadic LPA | 0.17(-0.20,0.53) | 0.375 | 0.28(-0.09,0.65) | 0.132 | 0.24(-0.18,0.66)^d^ | 0.259 |

**Table S1** Linear regression of the associations between PA, SB variables, and HGS

PA, physical activity; SB,sedentary behaviour; HGS, hand grip strength; B, regression coefficient; CI, confidence intervals;

MVPA, moderate-to-vigorous-intensity physical activity; LPA, light intensity physical activity; Bouted, duration≥10 min; Sporadic, duration＜10 min.

The superscripts ^a,^ ^b,^ ^c, d,^ and d represent Model 3a, Model 3b, Model 3c, and Model 3d;

Model 1: Adjusting accelerometer daily wear time and age;

Model 2: Model 1+BMI, Living alone, Income, Number of chronic diseases, AIS score, MNA score, and MMSE score;

Model 3a: Model 2+total MVPA time;

Model 3b: Model 2+total SB time;

Model 3c: Model 2+total SB time, and additionally adjusted for bouted MVPA and sporadic MVPA to each other;

Model 3d: Model 2+total MVPA time, and additionally adjusted for bouted LPA and sporadic LPA to each other.

**Table S2** Linear regression of the associations between PA, SB variables, and OLSTEC

| The PA and SB variables | B (95 % CI) | | | | | |
| --- | --- | --- | --- | --- | --- | --- |
|  | Model 1 | P | Model 2 | P | Model 3 | P |
| Per 60 min increment of |  |  |  |  |  |  |
| Total SB time | -0.23(-0.70, 0.24) | 0.331 | -0.20(-0.67, 0.28) | 0.422 | -0.13(-0.65, 0.39)^a^ | 0.632 |
| 30-min bout of SB | -0.38(-0.82,0.07) | 0.095 | -0.29(-0.74,0.16) | 0.205 | -0.25(-0.72,0.21)^a^ | 0.283 |
| 60-min bout of SB | -0.46(-1.10,0.17) | 0.149 | -0.35(-0.99,0.28) | 0.267 | -0.31(-0.96,0.33)^a^ | 0.344 |
| Per 10 min increment of |  |  |  |  |  |  |
| Total MVPA time | 0.88(0.00.1.76) | 0.049* | 0.42(-0.48,1.33) | 0.361 | 0.33(-0.66,1.32)^b^ | 0.518 |
| Bouted MVPA | 0.16(-0.27, 0.58) | 0.472 | 0.01(-0.44,0.43) | 0.978 | -0.09(-0.54,0.36)^c^ | 0.693 |
| Sporadic MVPA | 0.27(0.01,0.53) | 0.04* | 0.17(-0.09,0.43) | 0.202 | 0.19(-0.08,0.46)^c^ | 0.159 |
| Per 30 min increment of |  |  |  |  |  |  |
| Total LPA time | 0.06( -0.19,0.31) | 0.631 | 0.08(-0.18,0.34) | 0.544 | 0.06(-0.20,0.32)^a^ | 0.632 |
| Bouted LPA | -0.02( -0.25,0.21) | 0.867 | 0.02(-0.21,0.26) | 0.861 | 0.09(-0.17,0.36)^d^ | 0.484 |
| Sporadic LPA | 0.82( 0.04,1.60) | 0.04* | 0.52(-0.28,1.31) | 0.202 | 0.62(-0.29,1.52)^d^ | 0.182 |

PA, physical activity; SB,sedentary behaviour; OLSTEC, One-legged stance test with eyes closed; B, regression coefficient; CI, confidence intervals;

MVPA, Moderate-to-vigorous-intensity physical activity; LPA, light intensity physical activity; Bouted, duration≥10 min; Sporadic, duration＜10 min.

The superscripts ^a,^ ^b,^ ^c, d,^ and d represent Model 3a, Model 3b, Model 3c, and Model 3d;

Model 1: Adjusting accelerometer daily wear time and age;

Model 2: Model 1+BMI, Living alone, Income, Number of chronic diseases, AIS score, MNA score, and MMSE score;

Model 3a: Model 2+total MVPA time;

Model 3b: Model 2+total SB time;

Model 3c: Model 2+total SB time, and additionally adjusted for bouted MVPA and sporadic MVPA to each other;

Model 3d: Model 2+total MVPA time, and additionally adjusted for bouted LPA and sporadic LPA to each other; *P-value<0.05

**Table S3** Linear regression of the associations between PA, SB variables, and UWS

| The PA and SB variables |  |  | B (95 % CI) |  |  |  |
| --- | --- | --- | --- | --- | --- | --- |
|  | Model 1 | P | Model 2 | P | Model 3 | P |
| Per 60 min increment of |  |  |  |  |  |  |
| Total SB time | 0.05(0.26, 0.08) | <0.001** | 0.05(0.02, 0.08) | <0.001** | 0.01(-0.02, 0.04)^a^ | 0.515 |
| 30-min bout of SB | 0.03(0.01,0.06) | 0.015* | 0.02(-0.00,0.05) | 0.052 | 0.00(-0.03,0.03)^a^ | 0.894 |
| 60-min bout of SB | 0.04(0.00,0.08) | 0.037* | 0.03(-0.00,0.07) | 0.081 | 0.01(-0.03,0.04)^a^ | 0.765 |
| Per 10 min increment of |  |  |  |  |  |  |
| Total MVPA time | -0.23(-0.28,-0.18) | <0.001** | -0.20(-0.25,-0.15) | <0.001** | -0.19(-0.25,-0.14)^b^ | <0.001** |
| Bouted MVPA | -0.09(-0.11,-0.06) | <0.001** | -0.08(-0.10,-0.05) | <0.001** | -0.07(-0.10,-0.04)^c^ | <0.001** |
| Sporadic MVPA | -0.02(-0.03,0.00) | 0.056 | -0.01(-0.02,0.01) | 0.387 | -0.00(-0.02,0.01)^c^ | 0.706 |
| Per 30 min increment of |  |  |  |  |  |  |
| Total LPA time | -0.01(-0.03,0.00) | 0.101 | -0.01(-0.03,0.00) | 0.091 | -0.01(-0.02,0.01)^a^ | 0.515 |
| Bouted LPA | -0.01(-0.02,0.01) | 0.354 | -0.01(-0.02,0.00) | 0.200 | -0.00(-0.02,0.01)^d^ | 0.551 |
| Sporadic LPA | -0.05(-0.09,0.00) | 0.056 | -0.02(-0.07,0.03) | 0.387 | 0.00(-0.05,0.05)^d^ | 0.967 |

PA, physical activity; SB, sedentary behaviour; UWS, usual walking speed; B, regression coefficient; CI, confidence intervals;

MVPA, moderate-to-vigorous-intensity physical activity; LPA, light intensity physical activity; Bouted, duration≥10 min; Sporadic, duration＜10 min.

The superscripts ^a,^ ^b,^ ^c, d,^ and d represent Model 3a, Model 3b, Model 3c, and Model 3d;

Model 1: Adjusting accelerometer daily wear time and age;

Model 2: Model 1+BMI, Living alone, Income, Number of chronic diseases, AIS score, MNA score, and MMSE score;

Model 3a: Model 2+total MVPA time;

Model 3b: Model 2+total SB time;

Model 3c: Model 2+total SB time, and additionally adjusted for bouted MVPA and sporadic MVPA to each other;

Model 3d: Model 2+total MVPA time, and additionally adjusted for bouted LPA and sporadic LPA to each other; *P-value<0.05, **P-value<0.01

**Table S4** Linear regression of the associations between PA, SB variables, and MWS

| The PA and SB variables | B (95 % CI) | | | | | |
| --- | --- | --- | --- | --- | --- | --- |
|  | Model 1 | P | Model 2 | P | Model 3 | P |
| Per 60 min increment of |  |  |  |  |  |  |
| Total SB time | 0.19(0.09, 0.29) | <0.001** | 0.18(0.08, 0.29) | <0.001** | 0.00(-0.02, 0.20)^a^ | 0.099 |
| 30-min bout of SB | 0.02(0.00,0.04) | 0.025* | 0.02(0.00,0.04) | 0.031* | 0.01(-0.01,0.03)^a^ | 0.510 |
| 60-min bout of SB | 0.02(-0.01,0.05) | 0.112 | 0.02(-0.01,0.05) | 0.128 | 0.00(-0.02,0.03)a | 0.765 |
| Per 10 min increment of |  |  |  |  |  |  |
| Total MVPA time | -0.15(-0.19,-0.11) | <0.001** | -0.12(-0.16,0.08) | <0.001** | -0.12(-0.16,-0.75)^b^ | <0.001** |
| Bouted MVPA | -0.05(-0.07,-0.03) | <0.001** | -0.04(-0.06,-0.02) | <0.001** | -0.03(-0.05,0.02)^c^ | <0.001** |
| Sporadic MVPA | -0.02(-0.03,-0.00) | 0.009** | -0.01(-0.02,0.00) | 0.222 | -0.01(-0.02,0.01)^c^ | 0.334 |
| Per 30 min increment of |  |  |  |  |  |  |
| Total LPA time | -0.00(-0.01,0.01) | 0.386 | -0.01(-0.02,0.00) | 0.103 | -0.00(-0.02,0.01)^a^ | 0.451 |
| Bouted LPA | -0.00(-0.01,0.01) | 0.985 | -0.01(-0.02,0.00) | 0.262 | -0.00(-0.02,0.01)^d^ | 0.421 |
| Sporadic LPA | -0.05(-0.08,-0.01) | 0.009** | -0.02(-0.06,0.01) | 0.222 | -0.01(-0.05,0.03)^d^ | 0.580 |

PA, physical activity; SB, sedentary behaviour; MWS, maximum walking speed; B, Regression Coefficient; CI, confidence intervals;

MVPA, moderate-to-vigorous-intensity physical activity; LPA, light intensity physical activity; Bouted, duration≥10 min; Sporadic, duration＜10 min.

The superscripts ^a,^ ^b,^ ^c, d,^ and d represent Model 3a, Model 3b, Model 3c, and Model 3d;

Model 1: Adjusting accelerometer daily wear time and age;

Model 2: Model 1+BMI, Living alone, Income, Number of chronic diseases, AIS score, MNA score, and MMSE score;

Model 3a: Model 2+total MVPA time;

Model 3b: Model 2+total SB time;

Model 3c: Model 2+total SB time, and additionally adjusted for bouted MVPA and sporadic MVPA to each other;

Model 3d: Model 2+total MVPA time, and additionally adjusted for bouted LPA and sporadic LPA to each other; *P-value<0.05, **P-value<0.01

**Table S5** Linear regression of the associations between PA, SB variables, and CT

| The PA and SB variables | B (95 % CI) | | | | | |
| --- | --- | --- | --- | --- | --- | --- |
|  | Model 1 | P | Model 2 | P | Model 3 | P |
| Per 60 min increment of |  |  |  |  |  |  |
| Total SB time | 0.19( 0.09, 0.29) | <0.001** | 0.18(0.08 ,0.29) | <0.001** | 0.09(-0.02, 0.20)^a^ | 0.099 |
| 30-min bout of SB | 0.19(0.09, 0.28) | <0.001** | 0.17(0.08,0.27) | <0.001** | 0.11(0.01,0.21)^a^ | 0.021* |
| 60-min bout of SB | 0.17(0.03,0.30) | 0.017* | 0.16(0.02,0.29) | 0.022* | 0.09(-0.05,0.23)^a^ | 0.197 |
| Per 10 min increment of |  |  |  |  |  |  |
| Total MVPA time | -0.61(-0.80,-0.42) | <0.001** | -0.51(-0.70,-0.32) | <0.001** | -0.44(-0.65,-0.23)^b^ | <0.001** |
| Bouted MVPA | -0.19(-0.28,-0.10) | <0.001** | -0.15(-0.24,-0.05) | 0.002* | -0.11(-0.20,-0.01)^c^ | 0.024* |
| Sporadic MVPA | -0.07(-0.13,-0.01) | 0.012* | -0.04(-0.10,-0.01) | 0.150 | -0.04(-0.10,0.02)^c^ | 0.153 |
| Per 30 min increment of |  |  |  |  |  |  |
| Total LPA time | -0.06(-0.11,-0.00) | 0.035* | -0.07(-0.12,-0.01) | 0.018* | -0.05(-0.10,0.01)^a^ | 0.099 |
| Bouted LPA | -0.03(-0.08,-0.02) | 0.238 | -0.04(-0.09,0.01) | 0.084 | -0.05(-0.11,0.00)^d^ | 0.073 |
| Sporadic LPA | -0.22(-0.38,-0.05) | 0.012* | -0.12(-0.29,0.04) | 0.150 | -0.13(-0.32,0.06)^d^ | 0.175 |

PA, physical activity; SB, sedentary behaviour; CT, chair-standing time; B, regression coefficient; CI, confidence intervals;

MVPA, moderate-to-vigorous-intensity physical activity; LPA, light intensity physical activity; Bouted, duration≥10 min; Sporadic, duration＜10 min.

The superscripts ^a,^ ^b,^ ^c, d,^ and d represent Model 3a, Model 3b, Model 3c, and Model 3d;

Model 1: Adjusting accelerometer daily wear time and age;

Model 2: Model 1+BMI, Living alone, Income, Number of chronic diseases, AIS score, MNA score, and MMSE score;

Model 3a: Model 2+total MVPA time;

Model 3b: Model 2+total SB time;

Model 3c: Model 2+total SB time, and additionally adjusted for bouted MVPA and sporadic MVPA to each other;

Model 3d: Model 2+total MVPA time, and additionally adjusted for bouted LPA and sporadic LPA to each other; *P-value<0.05, **P-value<0.01

**Table S6** Linear regression of the associations between PA, SB variables, and Z-score

| The PA and SB variables | B (95 % CI) | | | | | |
| --- | --- | --- | --- | --- | --- | --- |
|  | Model 1 | p | Model 2 | p | Model 3 | p |
| Per 60 min increment of | | | | | | |
| Total SB time | -0.29(-0.44, -0.14) | <0.001** | -0.29(-0.44, -0.14) | <0.001** | -0.09(-0.25, 0.07)^a^ | 0.266 |
| 30-min bout of SB | -0.27(-0.41, -0.13) | <0.001** | -0.24(-0.38, -0.10) | 0.001** | -0.12(-0.26, 0.02)^a^ | 0.093 |
| 60-min bout of SB | -0.29(-0.50, -0.09) | 0.005** | -0.29(-0.48, -0.09) | 0.005** | -0.13(-0.33, 0.07)^a^ | 0.202 |
| Per 10 min increment of | | | | | | |
| Total MVPA time | 1.18(0.90, 1.45) | <0.001** | 1.00(0.73, 1.28) | <0.001** | 0.94(0.63, 1.23)^b^ | <0.001** |
| Bouted MVPA | 0.38(0.25, 0.52) | <0.001** | 0.31(0.17, 0.44) | <0.001** | 0.25(0.11, 0.39)^c^ | <0.001** |
| Sporadic MVPA | 0.14(0.06, 0.22) | 0.001** | 0.09(0.00, 0.17) | 0.042* | 0.08(-0.00, 0.17)^c^ | 0.065 |
| Per 30 min increment of | | | | | | |
| Total LPA time | 0.07(-0.01, 0.15) | 0.079 | 0.09(0.01, 1.17) | 0.037* | 0.05(-0.03,0.13)^a^ | 0.266 |
| Bouted LPA | 0.02(-0.05, 0.10) | 0.521 | 0.05(-0.02, 0.12) | 0.195 | 0.05(-0.03, 0.14)^d^ | 0.193 |
| Sporadic LPA | 0.42(0.17, 0.67) | 0.001** | 0.26(0.01, 0.51) | 0.042* | 0.20(-0.08,0.48)^d^ | 0.158 |

PA, physical activity; SB, sedentary behaviour; B, regression coefficient; CI, confidence intervals;

MVPA, moderate-to-vigorous-intensity physical activity; LPA, Light intensity physical activity; Bouted, duration≥10 min; Sporadic, duration＜10 min.

The superscripts a, b, c, d, and d represent Model 3a, Model 3b, Model 3c, and Model 3d;

Model 1: Adjusting accelerometer daily wear time and age;

Model 2: Model 1+BMI, Living alone, Income, Number of chronic diseases, AIS score, MNA score, and MMSE score;

Model 3a: Model 2+total MVPA time;

Model 3b: Model 2+total SB time;

Model 3c: Model 2+total SB time, and additionally adjusted for bouted MVPA and sporadic MVPA to each other;

Model 3d: Model 2+total MVPA time, and additionally adjusted for bouted LPA and sporadic LPA to each other. *P-value<0.05, **P-value<0.01
